# Maya Postclassic persistence in the Birds of Paradise Wetland Fields, Belize

**DOI:** 10.1073/pnas.2521892123

**Published:** 2026-03-02

**Authors:** Lara M. Sánchez-Morales, Timothy P. Beach, Sheryl Luzzadder-Beach, Samantha Krause, Duncan Cook, Byron Smith, David Lentz, Carlos Quiroz, William Pratt, Lori Phillips, Thomas Guderjan, Colleen Hanratty, Fred Valdez

**Affiliations:** ^a^Department of Anthropology, New York University, New York, NY 10012; ^b^Department of Geography and the Environment, University of Texas at Austin, Austin, TX 78712; ^c^Department of Geography and Environmental Studies, Texas State University, San Marcos, TX 78666; ^d^School of Arts and Humanities, Australian Catholic University, Banyo, QLD 4014, Australia; ^e^Department of Anthropology, California State Polytechnic University Humboldt, Arcata, CA 95521; ^f^Department of Biological Sciences, University of Cincinnati, Cincinnati, OH 45221; ^g^Belize Ministry of Education, Culture, Science and Technology, Belize City, Belize; ^h^Acacia Heritage Consulting, Austin, TX 78731; ^i^Department of Social Sciences, University of Texas at Tyler, Tyler, TX 75799; ^j^Department of Anthropology, University of Texas at Austin, Austin, TX 78712

**Keywords:** ancient Maya, Terminal Classic to Postclassic persistence, wetland fields, socioenvironmental resilience, ancient wood posts

## Abstract

Rapid decay of organic artifacts obscures our view of ancient tropical settlements. We present a study of architecture and artifact assemblages from an ancient settlement excavated at the Birds of Paradise (BOP) fields, the largest lidar and field-validated ancient Maya wetland field complex. The site’s unique setting preserved architectural wood and provides exceptional evidence of Maya Terminal Classic (CE 800 to 1000) to Postclassic (CE 1000 to 1500) persistence within tropical wetlands through periods of large-scale societal and climate change. A new Bayesian chronological model tightens the occupation history for the broader BOP wetlands based on 14 new radiocarbon dates of wood and charcoal from this settlement. These wetlands and their preserved ancient wood face imminent destruction through modern land uses.

## Significance

This research provides compelling evidence for ancient Maya adaptation to the profound challenges experienced during the Terminal Classic (CE 800 to 1000) to the Postclassic (CE 1000 to 1500) through the use of wetland agroecosystems. As large urban centers across the Maya regions succumbed to interconnected socioenvironmental factors, communities at the Birds of Paradise complex persisted through this transition by erecting a series of raised earthen, stone, and wood structures with direct access to the copious resources and connectivity afforded by this riverine wetland system. Our long-term study of this landscape and a recently uncovered settlement with uniquely preserved wooden architecture and domestic remains support urgent calls for wetland conservation in the race against modern climate change and land use.

**Fig. 1.**
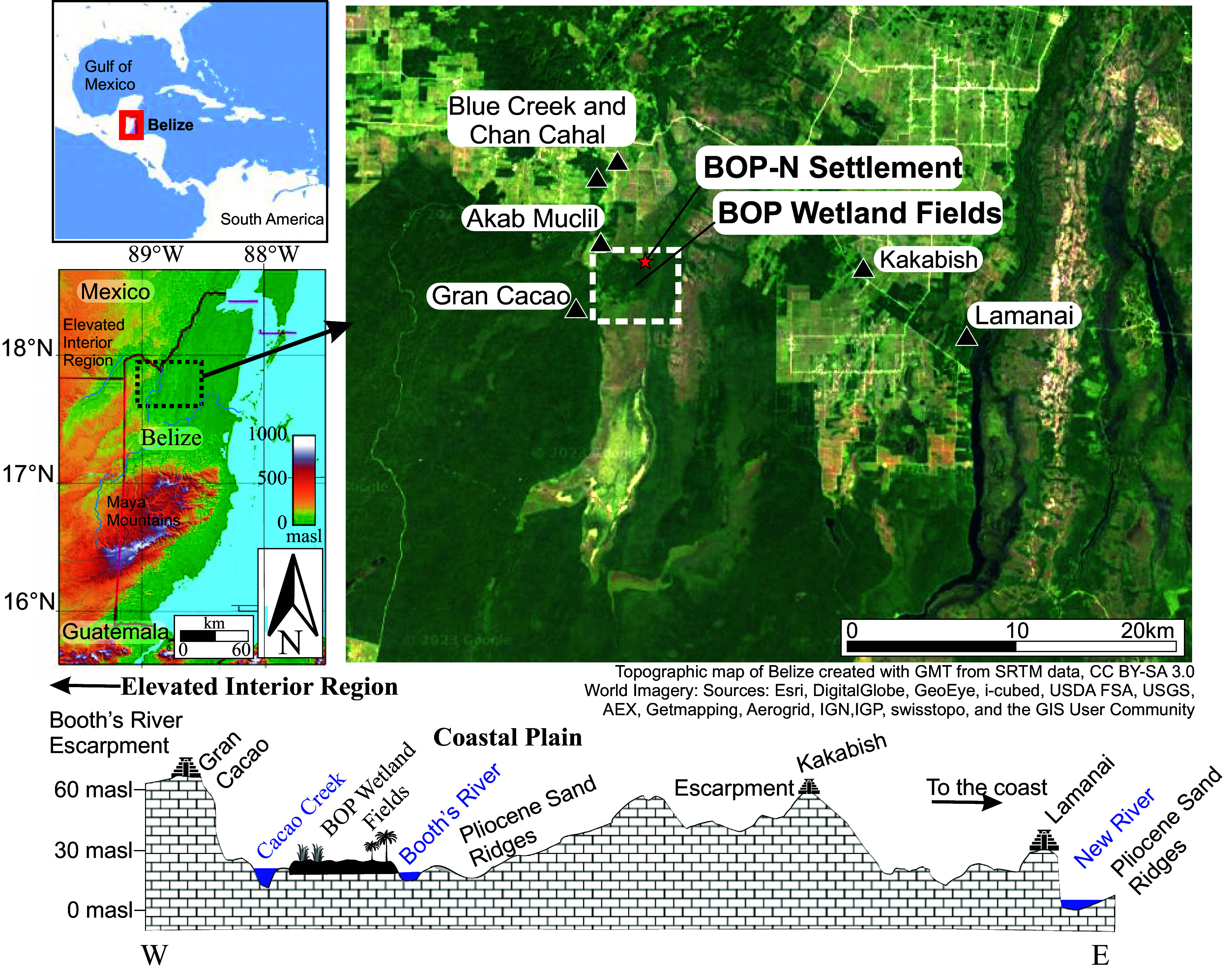
Study area. Northern Belize Maya sites and BOP wetlands plan view (*Upper*) and elevation profile and site landscape placement (*Lower*).

Ancient cultures—like modern ones—relied on wetlands for ecosystem services, using them for hunting, fishing, and gathering through the transition of agricultural domestication to intensive farming. Wetlands also provided, and still provide, refuge during periods of drought and societal upheaval (1–4). There is ample evidence demonstrating that ancient cultures lived sustainably for millennia with their wetlands. Yet today, wetland destruction is occurring rapidly around the world, causing enormous losses to ecosystem services as well as destruction of the Indigenous knowledge of sustainable ancient wetland farming systems that can provide vital lessons for adaptation to the modern climate crisis (5). In the tropical lowlands of Mesoamerica, the earliest evidence for agriculture, a key form of indigenous knowledge, dates to 5000 BCE (*SI Appendix*, Table S1) in the wetlands of Tabasco (6), Campeche, Mexico (7), and Belize (8). Thus, evidence for wetland farming in the Maya Lowlands precedes even the Maya Preclassic Period (9, 10), and some studies from parts of the Maya Lowlands indicate wetland farming persisted through the Classic (CE 550 to 900) and, sporadically, into the Maya Postclassic (CE 1000 to 1500) (11–13) (*SI Appendix*, Table S1). Other studies from the same areas disagree, maintaining wetland farming persisted through the Maya Classic (14) while another highly cited article from the region with no subsequent research contends that these systems disintegrated long before the Maya Terminal Classic “collapse” (CE 800 to 1000) (10). Here, we present multiple findings that indicate the continued use of wetland agroecosystems that persisted through the Terminal Classic “Collapse” (800 to 1000 CE) and well beyond into the Postclassic (1000 to 1500 CE). Moreover, this wetland cultural persistence occurred within a kilometer of uplands (~100 m higher) where abandonment was widespread in the Terminal Classic. Previous studies provide a timeline of depopulation and the end of intensive agriculture in these adjacent upland sites of the Elevated Interior Region (EIR) of the central Maya Lowlands (Fig. 1) during the Terminal Classic, which is followed by cultural and economic changes, migration, and a shift toward coastal resources (15–18).

This study derives from new excavations and multiple proxy data from the Birds of Paradise (BOP) wetland field complex in northwestern Belize (19–22). Here, we also present findings of unusually well-preserved wood in a Maya settlement that connects spatially and chronologically with an intensive wetland agricultural system. The settlement includes a main platform and surrounding activity areas with ten well-preserved wood posts that make up the site’s structural foundations. The wood posts within the platform and the activity areas help us establish a radiocarbon chronology of human occupation, provide rare information about ancient forest resource use (23), and suggest that other Maya wetland sites may contain this highly perishable building material. We hypothesize that this settlement and the surrounding wetland complex exemplify persistent places (2–5, 13, 24, 25), which endured even as surrounding areas succumbed to multiple possible factors for societal unraveling such as drought, warfare, and rerouted trade networks during the Maya Terminal Classic Collapse (24, 26–30).

Because of the poor preservation of Maya structures beyond stone-built urban centers, few studies have demonstrated wetland farming at the settlement scale that surrounds the urban footprint (25). Here, we show the expanded footprint of Maya urbanscapes and wetland agriculture that integrated with and extended beyond urban places. This study brings together multiple lines of evidence that indicate reliance on cultivating wetlands during and through key societal transitions. This site lies in a transition zone at the foot of the Elevated Interior Region (EIR), which experienced cultural fluorescence during the Classic but abandonment in the Postclassic, and the eastern coastal plains, where some trade and power continued and redeveloped during the Postclassic (29). At this wetland settlement, against the headwinds of broader ancient Maya societal transformation (31), we show wetland and community persistence, resilience, and sustainability. This evidence includes a new Bayesian radiocarbon chronology and evidence for site use through ceramic, lithic, and faunal assemblages that indicate regional and long-range connectivity. We also reconstruct the site’s formation history through sedimentological analyses. This study provides a unique view of life in the Maya Postclassic in this little studied transitional zone that connects the EIR with the coastal environments and populations to the east.

The BOP wetland field complex (Fig. 1) is the largest lidar mapped and ground-verified contiguous system of ancient Maya wetland canals and fields, spanning 5 km^2^ with another 8 km^2^ nearby (20). This perennially moist riparian system lies mostly in the Rio Bravo Conservation and Management Area, which preserves wetlands, tropical forests, and cultural heritage in a region of rapid wetland losses and deforestation (32). Our knowledge of these ancient Maya wetland agroecosystems comes from high-resolution lidar (laser imaging, detection, and ranging) mapping, more than 20 excavations of prehispanic canals and fields, radiocarbon dating, artifacts, elemental and isotopic geochemistry, water chemistry, pollen, macrobotanical, charcoal, and phytolith analysis (20). Previous research here shows the ancient Maya long-practiced agriculture, with palynological evidence for crops including maize, cassava, arrowroot, beans, squash, and fruits. Paleoenvironmental records extracted from ancient fields and canals in the BOP wetlands also provide insights into ancient Maya field management strategies such as burning and water control (20, 33).

Previous radiocarbon dating indicates that the wetland field systems were in use for ~1,500 y, from the Late Preclassic to the Early Postclassic (~100 BCE to perhaps CE 1400) (13). In contrast, the Maya centers that flank the BOP wetlands (Gran Cacao and Akab Muclil) provide evidence for only sporadic Postclassic use (13). The timeline for Maya management of the BOP wetlands overlaps two extended regional droughts—the Late Preclassic to Early Classic (CE 200 to 300) and the Terminal Classic to the Early Postclassic (CE ca. 800 to 1000), though there is regional climatic variability, and the study area is 200 and 350 km away from the best resolved paleoclimate studies (34–37). If these droughts indeed impacted the wetland fields, their nearness to the perennial water table would have provided the most reliable water source during these periods of aridity (21).

The newly identified Maya settlement (BOP-N) associated with the BOP agroecosystem lies between two large ancient urban centers: Blue Creek (31), ~5 km to the northwest, and Gran Cacao (38), ~3.5 km to the southwest of the northern extent of the BOP wetlands (Fig. 1). Blue Creek, atop the Rio Bravo escarpment, was active from at least the Early Middle Preclassic (c. 800 BCE) to the Terminal Classic (CE 850 to 1000) and controlled adjacent wetland field systems at Chan Cahal (25). Gran Cacao lies atop the Booth’s River escarpment, where occupation spanned the Late Preclassic (400 BCE–CE 250) to the Terminal Classic (CE 850 to 1000) with visitation and some reoccupation during the Postclassic. Akab Muclil is a small site 2.5 km west of the BOP-N settlement and has multiproxy evidence of occupation, architectural modification, and maize farming into the Postclassic (39). Akab Muclil was likely an outlying node of public architecture and power for the Blue Creek polity.

**Fig. 2.**
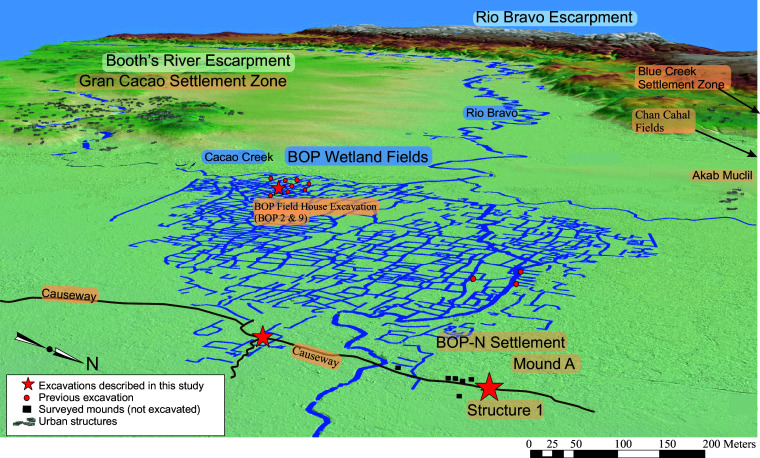
Drawing of oblique lidar DEM with key geographic features, Maya urban sites, and the BOP ancient wetland canals, causeway, and BOP-N settlement site.

**Fig. 3.**
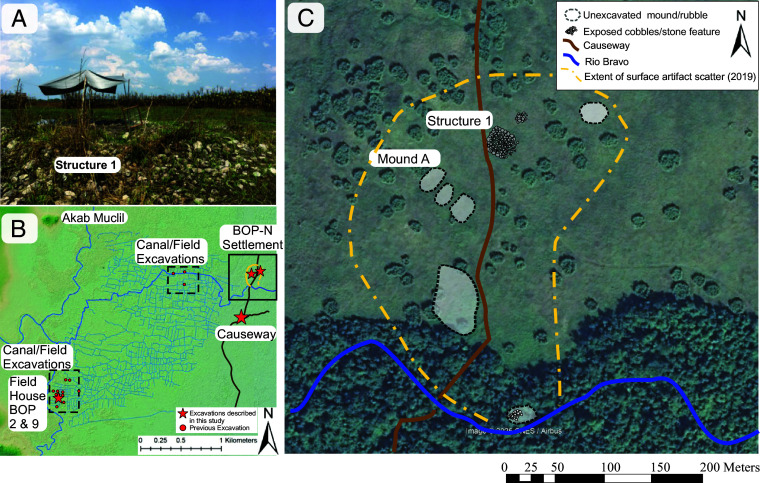
View of Structure 1 excavation set up (2018) (*A*). Location of BOP-N settlement relative to other urban sites, the causeway, wetland system, and excavations discussed in the text (*B*). Location of Structure 1, Mound A, causeway, and other surveyed mounds relative to the Rio Bravo (*C*).

Lidar surveys conducted in 2016 (20) and 2022 by the Northwestern Belize Lidar Consortium showed a linear feature that runs along the north–south axis, demarcating the eastern periphery of the BOP wetland system (Fig. 2). Mapping on the ground and from lidar and subsequent field survey and excavation indicated the feature was an ancient Maya causeway, ~2 m high, ~4 m wide, and at least 2 km long. This causeway may have provided access to the seasonally inundated fields and for water management (13). Ground verification of this causeway started in 2017, which led to the serendipitous identification of the Maya settlement (BOP-N) at its northern end, including scattered surface artifacts, eight raised mounds (likely eroded structure pads for buildings), wood posts, and Structure 1, a large, main elevated platform (Fig. 3*C*).

## Results

### Sedimentology and Chronology.

**Fig. 4.**
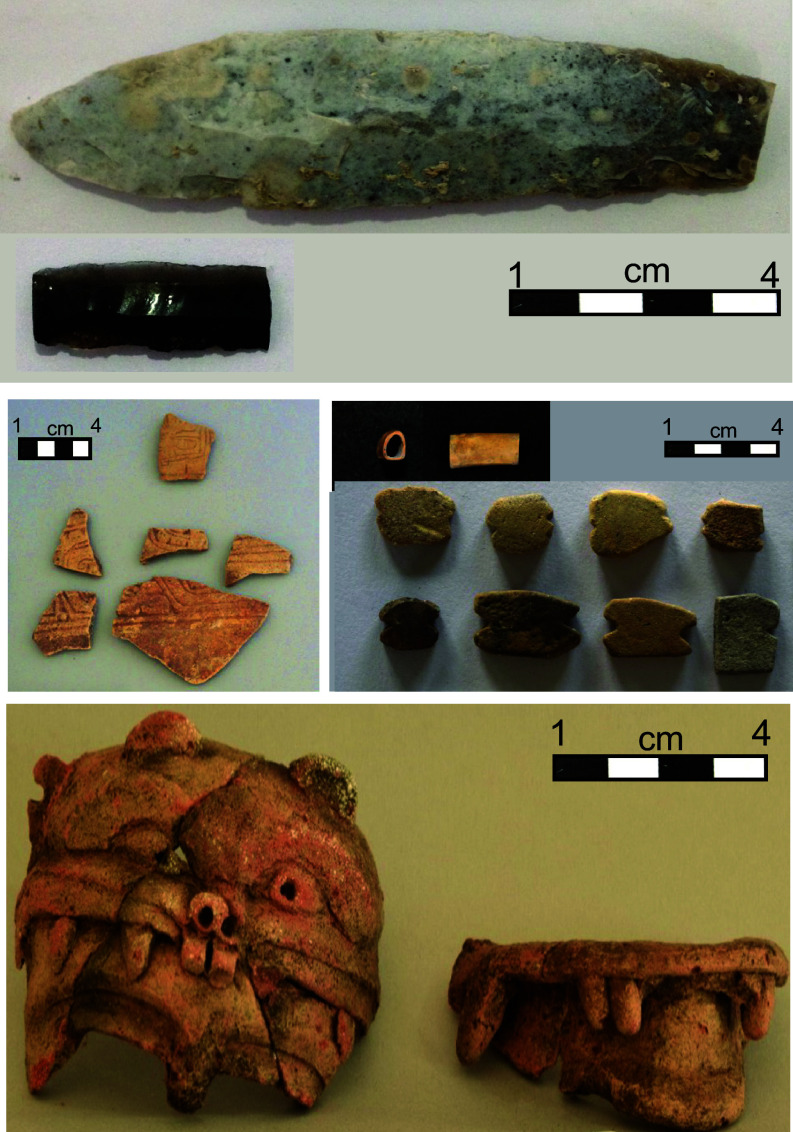
Examples from the artifact assemblage recovered from Structure 1 excavations. Lithic tools including obsidian blades (*Top*). Eroded vessel sherds, likely Pacmul Incised (*Center Left*) and Chen Mul Modeled censer (i.e., Jaguar head mask) (*Bottom*). Worked faunal remains and ceramic fishing net weights (*Middle Right*).

The causeway connecting the settlement and the wetland fields dates mainly to the Classic period. The causeway’s stratigraphy is composed of a 15-cm topsoil, 185 cm of compacted layers of loose, carbonate silt and fine sandy loam with intervening layers of sticky, organic-rich clay that hold the loose sediments together, and an organic and clay-rich paleosol at 2-m depth (Fig. 4). Three radiocarbon dates from the causeway sequence place the phases of construction between the Terminal Preclassic and the Early Classic. The sequence’s two lowermost dates came from charcoal, one from the top of the underlying paleosol at 2-m depth (ICA-18C/0117, CE 260 to 535, median age CE 410), and the other from the bottom of the organic clay fill at 1.6-m (ICA-18C/0116, CE 225 to 390, median age CE 305). The youngest date came from the organic sediment fill itself at 1.3-m depth (ICA-14C-7668, CE 415 to 565, median age CE 470). Once modeled together, the radiocarbon dates suggest that the causeway was constructed sometime after the most recent date in the Maya Classic and the older ages likely derive from deeper, older, quarried construction clay. There are no dates above 1.3 m, which may be a later level.

Mound A lay closest to the causeway and Structure 1, and we excavated this mound because of its large concentrations of cultural materials scattered across the surface, including a wide variety of lithic and ceramic artifacts, as well as embedded “jute” snail shells: a highly edible variety (*Pachychilus spp.*). The mound protruded 25-cm above the surrounding landscape and was excavated to a depth of 1.2-m below surface through a buried paleosol (or Ab horizon) devoid of artifacts. From 1.1 to 1.2-m depth is a very dark gray, organic-rich, clay-rich Ab horizon. Above this, the stratigraphy abruptly changed to a 70-cm thick layer of compacted, sterile, carbonate rich, fine sandy, and silty loam texture sediment, like the layers identified in the causeway (13). The top 50-cm of the mound is a deposit of unconsolidated, burned ceramic sherds, obsidian, and chert fragments, limestone grinding tools, and faunal material in a dark anthrosol (human modified soil) matrix rich in organic matter and charcoal (ref. 40: 170). Through the mound was an in-situ well-preserved wooden post (WP17-1), over 1 m long and ~10-cm in diameter, that was cut to a point at the base and ran vertically through the paleosol, carbonate sand, and activity layers protruding to the modern surface.

The chronology of Mound A was established from 2 charcoal fragments and by direct dating of the wood post. Charcoal from the top of the paleosol dated to the Terminal Classic to Early Postclassic transition (ICA-18C/0123, CE 900 to 1025, median age CE 995), while charcoal from the cultural layer at 35-cm dated to the Postclassic (ICA-18C/0122, CE 1200 to 1305, median age CE 1250). The age of the wood post (ICA-19W/01103, CE 990 to 1155, median age CE 1030) slightly overlaps the age of the paleosol, but the cross-cutting relationship of the post through the paleosol (and its date range) confirms that it should be younger than the paleosol.

Structure 1 is a limestone cobble-based earthen platform that protrudes 1.5 m above the surrounding surface. Excavations included an initial 1 × 2 m unit on the northeastern corner of the structure (2018) and then a 4 × 3 m macroblock in its southern part (2019). Excavations showed stratigraphy comparable to that found at Mound A but with additional investments in architectural engineering to raise the site higher in the landscape. Our 2018 excavation here yielded a wealth of artifacts, including ceramic vessels, lithic tools, faunal remains, and another well-preserved wooden post under a thick layer of cut limestone boulders. Although abundant, most of the excavated ceramics consist of highly eroded sherds or undiagnostic, unslipped jar body sherds. These ceramic types align with Lamanai traditions. Among the diagnostic sherds recovered, the ceramic assemblage indicates a chronological range spanning from the Late Classic (Tepeu 1 to 2) through the Terminal Classic (Tepeu 3) and into the Late Postclassic period. The Late and Terminal Classic components are represented by Tinaja Red: Variety Unspecified sherds (41). Among the Postclassic materials, notable pieces we recovered include one partially reconstructed Pozo unslipped bowl, one partially reconstructed Zakpah Orange-red: variety unspecified bowl, one partially reconstructed Zakbeeb Incised: variety unspecified bowl and several fragments from a Chen Mul Modeled censer dated from 1150 to 1450 (Fig. 4) (42–45).

**Fig. 5.**
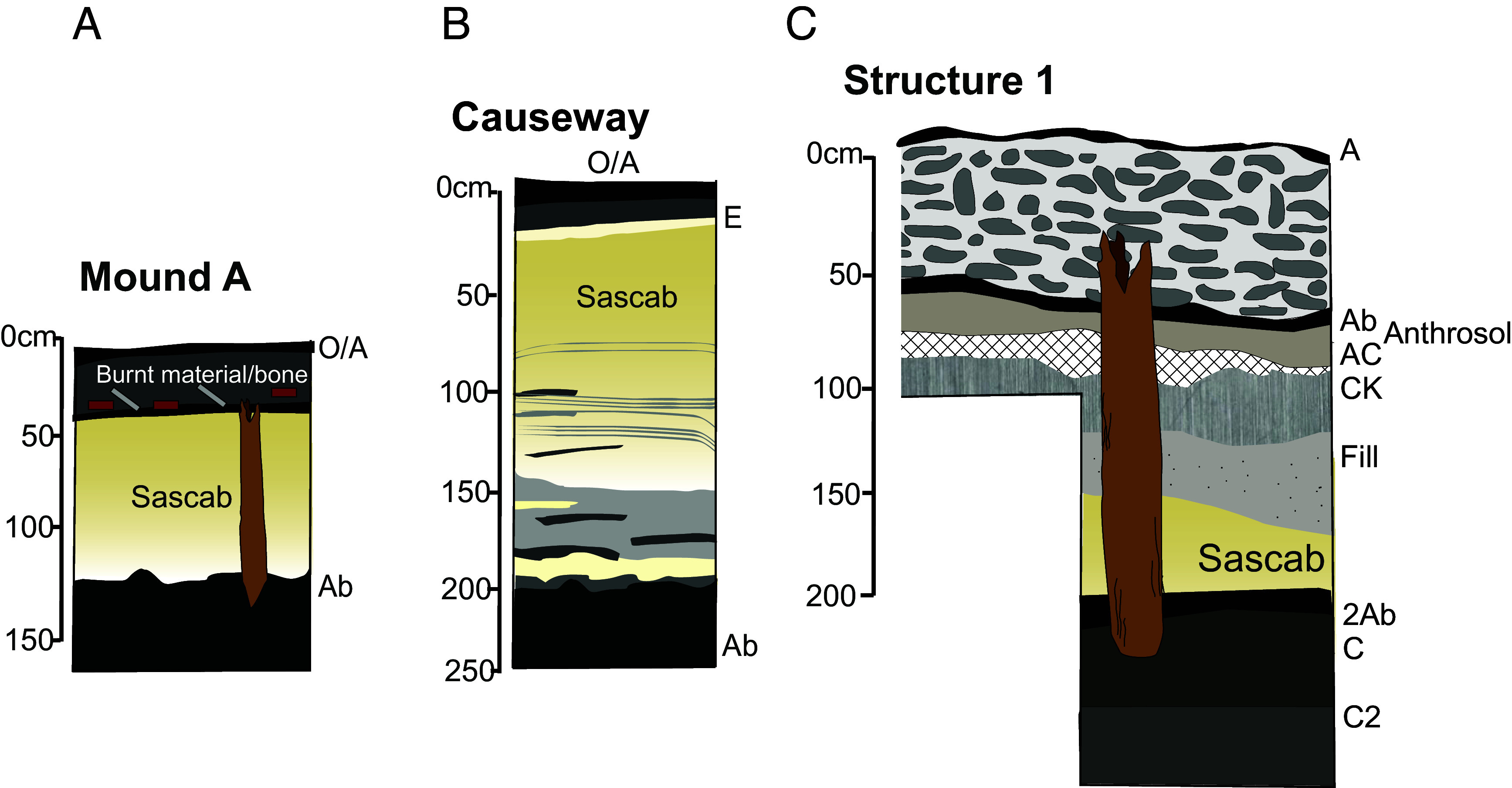
Stratigraphy from the excavation units described in the results. (*A*) Sequence of Mound A showing relationship to wood post (WP17-1); (*B*) stratigraphic sequence of the causeway; and (*C*) stratigraphic sequence of Structure 1 surrounding Post A.

**Fig. 6.**
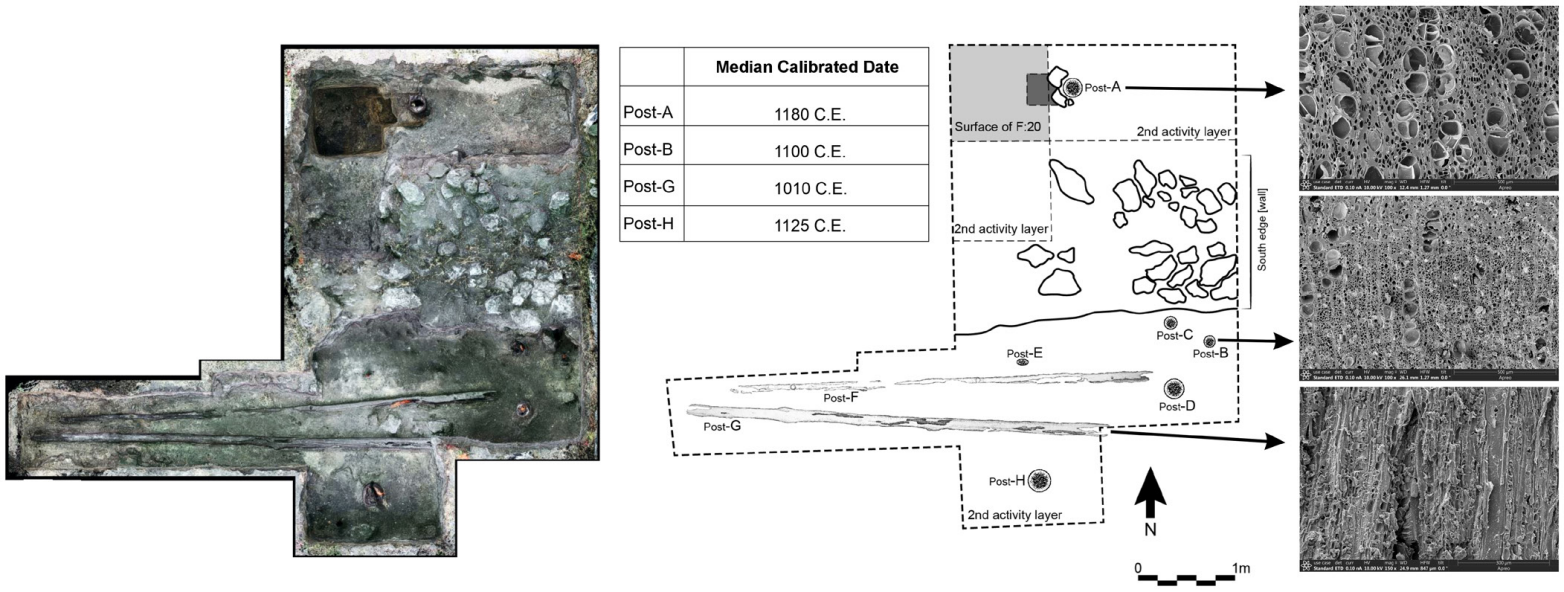
Plan view illustration of Structure 1 2019 macroblock excavation unit (dashed) with key features including uncovered wood posts with SEM microscans of wood tissue analyzed by Dr. David Lentz. [A, C-G: *Krugiodendrum ferreum*; B, H: *Exothea diphylla*].

Less than 5-cm below the modern topsoil, we found evidence of a daub floor and the top of the wall that built it up defined by a linear formation of limestone boulders and cobbles (Fig. 5). This limestone wall marked the extent of the upper edge of the platform structure and the final stone phase of construction. Excavations at the southern half of the structure ended above a buried anthropogenic A horizon that produced a high frequency of jute shells, other faunal remains, and charcoal within a sandy clay matrix. Here, we uncovered six vertical and two horizontal wood posts (Figs. 5 and 6, Posts A-G). Burned wood appeared in the hollow insides of the projecting posts. We estimate the posts on the northern side of the unit have a similar length based on their girth and similar context. The deepest excavation at the BOP-N settlement was in a 1 × 1 m unit in the western corner of the northern section, which reached 2.5 m and exposed the entire stratigraphy surrounding Post A (Figs. 5*C* and 6). This post was 184 cm long with an average diameter of 25 cm at the base. A portion of it lay beneath the limestone rubble. Right below this layer, we identified the same anthropogenic surface from the southern section. This anthrosol sequence had a 9 cm-thick, black A horizon formed in sandy loam sediments (AC/C horizons). Below this Postclassic anthrosol was a 30-cm-thick Ck horizon of hardened tufa, a carbonate precipitate formed over time in the seasonally moist root zone, which encased a diversity of faunal and botanical remains, ceramics, and lithics. This taphonomic event lay on top of another anthropogenic fill layer of light brownish gray color and sandy clay texture containing abundant and large charcoal pieces. Below the hardened tufa layer, we found a thick layer of pale brown, compacted carbonate silt and fine sandy loam similar to the sediment identified at both Mound A and the causeway. In the context of the platform, we found two layers of this carbonate sediment apparently held together by a fine, dark grayish brown layer of clayey texture sediment containing small charcoal fragments (Fig. 5*C*). These calcareous sediments lay abruptly on top of a well-developed, black, 34% organic matter, silty clay loam soil horizon (2Ab) that extended from 1.9 to 2.2-m depth.

Structure 1’s chronology is based on a total of 10 radiocarbon dates obtained from stratigraphic charcoal, peat, and wood. Three radiocarbon dates from the lowermost material (2Ab paleosol sequence) capture natural preconstruction deposition occurring from the Early to Terminal Classic Period. The lowermost date comes from organic soil at 2.20-m dated to CE 545 to 645 (ICA-14C-7084, median age CE 590). Organic sediments continued to aggrade here forming a peat sequence that generated dates of CE 670 to 875 (ICA-14C-7082, median age CE 740) at 2.09-m, and of CE 770 to 985 (ICA-14C-7083, median age CE 895) at 1.94-m. We interpreted this layer as the cumulic topsoil sequence of a wetland soil that had been aggrading based on its dated superposition and frequency of wetland obligatory *Pomaceae* snail shells. The abrupt boundary between this paleosol and the carbonate sediment above, along with numerous charcoal fragments suggests that the platform was constructed atop the paleosol after the Maya had cleared the area with fire. The next 14C dates in this sequence come from the construction phase of the platform itself confirming this process took place in the Maya Postclassic period (Fig. 5*C*). At 1.35-m, charcoal from fill material holding together two carbonate silt layers dated to CE 1165 to 1260 (ICA-19C/1270, median age CE 1215). Charcoal from the anthrosol above this (71-cm depth) radiocarbon dated to CE 1285 to 1400 (ICA-19C/1271, median age CE 1325).

**Fig. 7.**
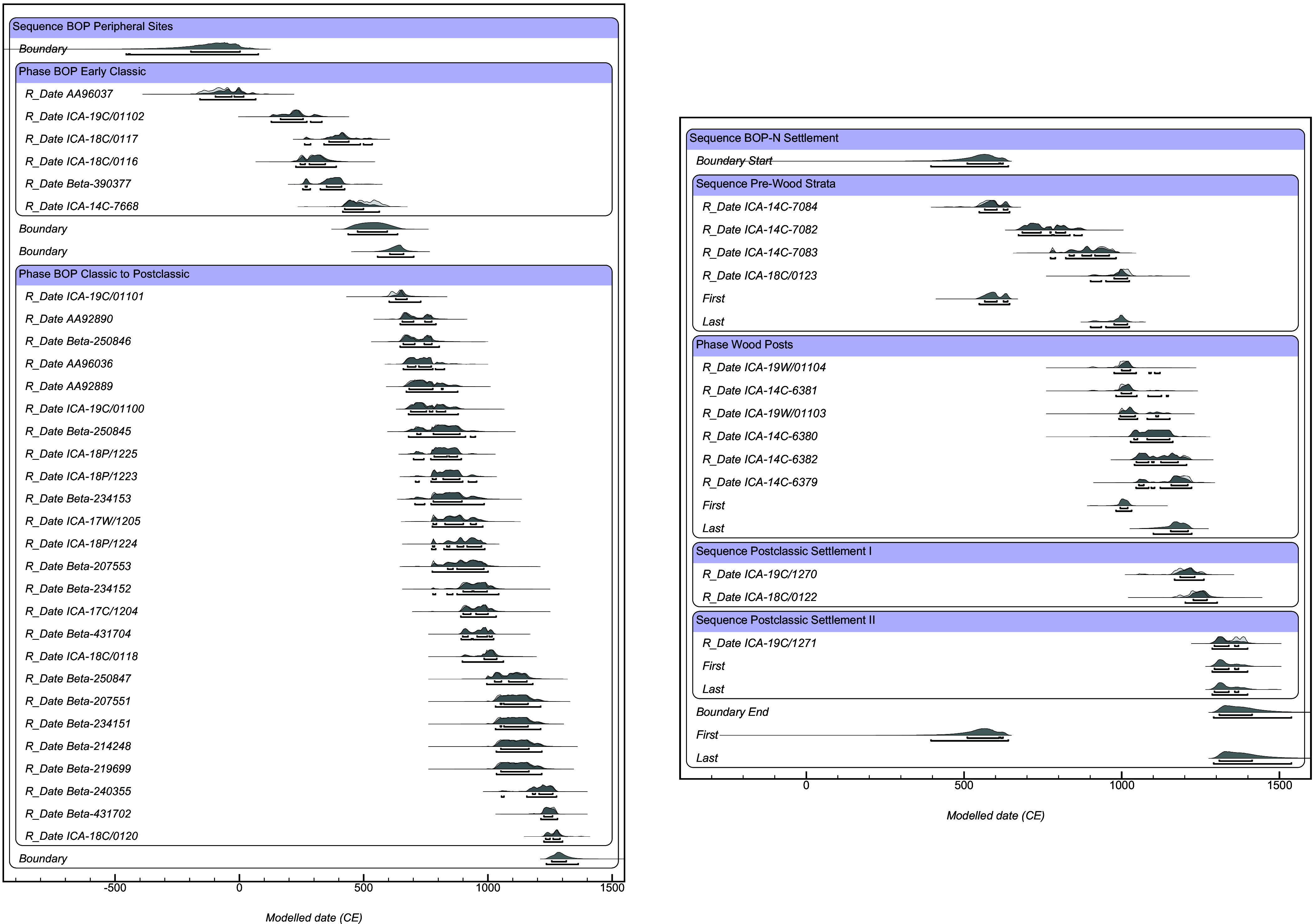
Modeled calendar ages (years CE) from the Bayesian radiocarbon model BZBOP25.

We radiocarbon dated one wood post from the 2018 excavation of Structure 1 and four wood posts from the 2019 excavations, which showed they have minimal differences in median calendar ages (<85 y). The modeled calendar ages estimated for these 5 posts overlap at 95.4% ranges (modeled beginning of wood use: CE 980 to 1035, end of wood use: CE 1100 to 1225, Fig. 7). The wood post from the 2018 excavations dated to CE 975 to 1125 (ICA- 19W/01104, median CE 1015). Two of the vertical posts found on the southern edge of the platform (2019 excavation) dated to CE 1025 to 1165 (PW-19-B: ICA-14C-6380, median CE 1100) and CE 1040 to 1205 (PW-19-H: ICA-14C-6382, median CE 1120). One of the horizontally placed posts (PW-19-G) dated to CE 980 to 1150 (ICA-14C-6381, median CE 1020). Post A, which was excavated to its maximum depth on the northern half of the unit, dated to CE 1045 to 1225 (ICA-14C-6379, median CE 1170). The use of wood posts is in the earliest period of this site’s history (median ages from the 11th and 12th century CE). The radiocarbon chronology for the platform suggests that construction began in the early Postclassic period and then the site was in use for various activities (discussed below) until the 14th century CE.

### Wood Use.

**Fig. 8.**
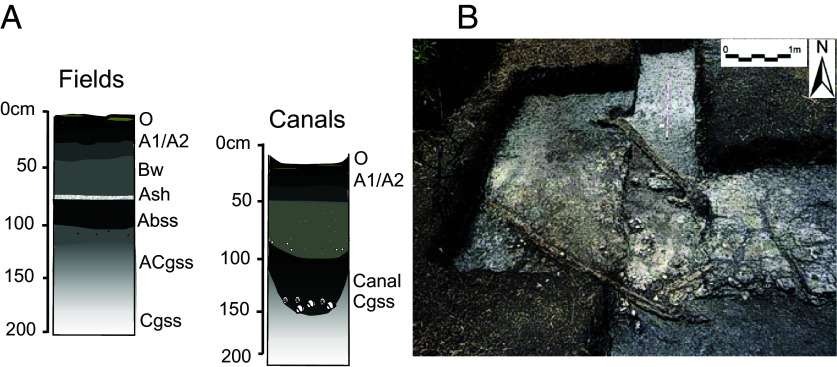
Stratigraphy of BOP canals and fields (*A*) and preserved wood and daub artifacts identified at wetland canal units (BOP 2 and 9) in 2013 (*B*).

Our 2006 excavations at BOP 2 and 9 yielded preserved wood (20). The wood here included a Maya digging stick, an adzed wood log that bridged a canal, more than one hundred wattle and daub fragments from a field house, and two preserved wood poles in canal excavations (Fig. 8). Together these produced Terminal to Postclassic and historical ages: The poles gave an age from the Early Postclassic period, CE 990 to 1160 (Beta-250847; median calendar age 1090 CE, *SI Appendix*, Tables S1 and S2 and *Note* 3), the adzed wood dated to CE 1220 to 1385 (Beta-207552, median calendar age 1270 CE, *SI Appendix*, Tables S1 and S2) in the Postclassic period, and the carved digging stick dated to sometime after the mid-17th century CE (Beta-219413, median calendar age 1780 CE, *SI Appendix*, Tables S1 and S2), but with large age uncertainties due to the reversal/plateau in the 14C calibration curve (46).

Structure 1 presented here yielded nine wooden posts: two positioned horizontally (PW19-F and -G) and seven vertically (PW-18; PW19-A through -E, and -H). Six of the posts (including the two horizontal ones) were identified as *Krugiodendron ferreum* (Vahl) Urb. and two as *Exothea diphylla* (Standl.) Lundell (Fig. 6). Both hardwood species are endemic to the Yucatán Peninsula, although *E. diphylla* (“wayam cox”) has a more limited natural geographic distribution than *K. ferreum* (“ironwood”). Both trees are rare in today’s upland forests west of BOP but are more common in transition forests between uplands and *bajos* such as the floodplain where Structure 1 is located (Nicholas Brokaw pers. comm. 2020). The species *E. diphylla* is listed as *bajo* species around the Maya site of Calakmul (47), 90 km northwest of this study site. The vertical posts in the excavations likely served to stabilize the structure. The fact that the tops of the vertical posts, including that of the mound protruded through the modern surface and showed signs of charring and decomposition, suggest that these were greater in length during the site’s occupation. Both horizontal posts (PW19-F and -G) were 10-15-cm in diameter and 4.5 m in length, making them thinner and longer than the vertical posts. Thus, considering their length, girth, and position these may have had a different structural function, but their context did not allow us to determine this. We hypothesize these were used for the roof and/or walls since Postclassic structures commonly had roofs made from perishable materials like posts and palm thatch (48) (see p. 230).

### Resource Acquisition.

The artifacts and ecofacts in Structure 1—and similarly those recovered from Mound A—are consistent with an activity site where intensive processing for subsistence took place. We also recovered fishing net weights of variable sizes (Fig. 4). Our interpretation of resource acquisition activities is based on the recovery and prevalence of ceramic net sinkers, the faunal assemblage, and the lithic assemblage. Ceramic net sinkers were abundant, and we consider these a key finding in this context because they provide evidence linking the BOP wetlands to fishing activities, which are also evidenced by the faunal assemblage. While notched sherds of the type recovered from our excavations are considered more common in the Late Classic than in the Postclassic (49), we do not use their presence in this context to establish the chronology of the site.

The taphonomic conditions of the site also permitted excellent preservation of faunal remains (*SI Appendix*, Table S3 and *Note* 4). The disparate and numerous faunal remain fragments demonstrate a wide and diverse array of habitats exploited for protein sources by the Postclassic residents of BOP-N. Befitting this ecotonal wetland, 66% of the species identified were terrestrial and 34% aquatic. Among many others, these included armadillo (*Dasypus novemcinctus*), agouti (*Dasyprocta punctata*), paca (*Cuniculus paca and Caviomorpha* sp.), domesticated dog (*Canis familiaris*), gray fox (*Urocyon cinereoargenteus*), Baird’s tapir (*Tapirella bairdii*), peccary (*Tayassuidae spp*.), two deer species (*Odocoileus virginianus* and *Mazma sp.*) turkey (*Meleagris* sp.), and several species of turtle (*Trachemys sp., Dermatemys sp., Kinosternidae sp.,* and *Testudines sp.)* and iguana (*Iguanidae sp.*). The many fish, turtle, and edible jute mollusk remnants may indicate trapping and perhaps farming, which is well attested in Pre-Columbian societies, but little studied yet suspected in the Maya world (50). Over 80% of the total vertebrate assemblage shows signs of burning ranging from 1 to 4 on Stiner and Kuhn’s (51) burning color scale. Also, two large Aves long bone fragments had modifications related to tool or bead production (Fig. 4). One specimen features a cut end with polishing, while the other has both ends cut, smoothed, as prepared for bead production. Modifications of the invertebrate specimens include spire lopping and puncture holes on jute shells, modifications associated with processing for consumption (52, 53). Field identification of bone fragments from the mound’s 50-cm thick anthrosol included deer, bird (probably turkey), some small fish vertebrae, and hundreds of jute shells.

The platform and mound excavations also yielded a rich lithic assemblage that suggests the processing of diverse dietary sources, although it is possible that other materials such as fibers were also processed using these tools. Alongside the lithics, the diversity and abundance of faunal remains highlight the importance of hunting and fishing within this intensively farmed wetland.

## Discussion

The BOP-N settlement is a unique and important finding in the ongoing studies of ancient Maya agroecosystems for three major reasons introduced here and discussed in the next paragraphs. First, the BOP-N settlement provides evidence of persistent populations between the Elevated Interior Region (EIR) and the coastal regions during the Terminal Classic to Postclassic. While nearby upland urban centers in the EIR were abandoned, this population continued to emphasize wetland agriculture and provides our best evidence of other subsistence strategies such as fishing and gathering of other protein, reflected in the faunal assemblage. Second, this excavation has produced the largest assemblage of preserved wood in the Maya world beyond the coastal zone site of Paynes Creek, in southern Belize (23, 54–56). Third, the artifacts and ecofacts—faunal, lithics, ceramics, and wood — provide insight into the economy and subsistence during this period in these wetlands.

First, this settlement is a clear footprint of the Maya Postclassic in a region with little other evidence of persistence during this key period of Maya history, preceding European colonization. The spotty findings in 2006 of preserved wood and daub during BOP excavation sites 2 and 9 were a glimpse into a growing awareness that this region was an important focus for Maya occupation and farming for a long expanse of history, with land use that persisted through and beyond the much-contested “Classic Maya Collapse” into the Postclassic. It so happens that this clear Postclassic evidence occurs in wetlands, which lie close to the water table and thus would have afforded the Maya with the highest opportunity to adapt and persist through the droughts of the Terminal and Postclassic, as well as offering better conditions for preservation of perishable remains.

Radiocarbon dating and ceramic-based chronologies have provided a robust timeline for the human history of the BOP wetlands, which, along with the diverse artifacts and ecofacts, provide evidence for the intense and persistent economic and social value of wetland fields beyond typical Mesoamerican farming. Moreover, this growing evidence for Postclassic use of the wetlands extends the range and persistence for ancient farming of wetland areas to c. 4,000 y within a radius of 4 km around this site. Previous work showed a strong agricultural signal of maize and fruit trees coming from pollen evidence at Laguna Verde (4 km NW) starting in the Late Archaic (~4,500 y ago) (19), and direct polycultural use of the wetland field complex from as early as the Late Preclassic (~2,000 y ago) into the Postclassic (13, 20). At the nearby site of Akab Muclil, there is evidence of the Postclassic Maya continuing to invest in construction and maize agriculture (39) while other regional sites experience depopulation, discontinuous habitation, or simply pilgrimage (38). Taken together, these multiple lines of evidence indicate a persisting Terminal to Postclassic settlement focused on a central platform that was active on the northeast corner of the Ancient Maya BOP wetland fields, which had a key, raised access route along the wetland’s eastern margin and its cornucopia of resources.

Although abandonment occurred over adjacent urban centers like Blue Creek and Gran Cacao located far above easy access to water at the end of the Classic period with little or no evidence for subsequent reoccupation (38), other wetland sites persisted. The major persisting site is the monumental site of Lamanai (57), with its own wetland fields (58), next to the New River Lagoon, 24 km east of the BOP-N settlement and wetland field complex (Fig. 1). Other smaller examples include sites in northern Belize like the nearby Ka’kabish, Coco Chan (59, 60), and the Freshwater Creek watershed (61, 62). These sites had continuous habitation as early as the Late Preclassic (400 BCE–CE 250) (60) into the Postclassic Period with the highest population and extent during the 10th and 11th centuries CE (Terminal Classic–Early Postclassic) (60), monumental architectural construction in the Early Postclassic Period (57, 63, 64), to the Colonial Period (CE 1500 to 1700) (59), and had continued historical attention from the Spanish in the 1600s (65), the English in the 1800s (66), and even a home for 20th century refugees (67). Other areas such as sites in the Freshwater Creek area had Postclassic habitation whereas the wetland site of Kaxob had little evidence (see ref. 14: 155). The previous extensive excavations at BOP and the new findings from the BOP settlement together with the scattered evidence from Belize to Campeche’s Laguna de Terminos (68) suggest more widespread Postclassic uses and persistence across these coastal and riverine plains of the Maya world.

Second, particularly important at this site is architectural wood preservation, which is usually absent from the archaeological record of the Maya because of its perishable nature in tropical environments. The BOP-N platform offers a rare opportunity to study construction practices, as well as the distribution and use of local hardwood species during the Postclassic period. Other wood findings in the Maya world are scarce but have nonetheless contributed a great deal of information about Maya patterns of settlement and sourcing. The majority of Maya wood artifacts (including figurines, spears, and cache boxes) documented so far derived from cave contexts in Belize during the early 20th century (69) (and Gann as reported in McNatt 1996: 81) (70). The most recent significant discoveries of this type before our own was by Prufer and Dunham (2009) (71) in a cave in the Maya Mountains, and by McKillop (55, 56) on the seafloor in the Paynes Creek Salt Works in southern Belize. The latter produced 36 taxa of hardwoods and palmetto palms in 4042 wooden buildings (72) and different uses including a preserved paddle, which confirmed the connection between salt processing and mobility along waterways, associated with the Late to Terminal Classic (56). The excellent state of preservation of so much wood (and other ecofacts) in BOP-N is a result of the wetland’s anoxic conditions (73) and possibly the tufa layers that formed around materials because of the region’s carbonate-rich waters (74).

Third, the excavations presented here fuse traditional archaeology with geoarchaeological investigation of mound structures, wooden artifacts, and a linear causeway into a large wetland field complex. Through this approach, we have reconstructed segments of a tesserated anthropogenic landscape that demonstrates complex, resilient, and—most critically—long-lasting resource procurement during periods of societal transformation and climate change for the ancient Maya. As nearby urban places of power depopulated, the Maya remained at the BOP wetlands for subsistence, choosing to reinvest labor in maintaining and reworking these ancestral landscapes while coping with changing social, political, and environmental challenges. Surely, this reinvestment also led to resignification of identities and place-making for subsequent generations. As in other persistent places, this was clearly a long-term gathering place with hard infrastructure, with evidence of people processing and consuming food, goods, and community (75). We conclude that the archaeological, sedimentological, and paleoecological evidence shows that the BOP-N Structure and wetland field complex fits the model of a persistent place, and one that endured beyond the EIR’s collapse, and through periods of climate variability in the Maya Lowlands. We have presented evidence of long occupation, cultural materials, permanent shelter, and infrastructure connecting to other activity sites and shelter within the broader BOP wetland field complex. Our updated BOP chronology now shows Maya use of this wetland was underway as early as the 1st century BCE, and certainly by the 2nd century CE, with dated evidence of human activity across the wetlands continuing into the late 14th or perhaps early 15th century CE with no hiatus in the Late to Postclassic. In sum, this study provides quantitative, field-verified evidence for continued wetland agriculture and other wetland-based subsistence such as fishing in the Postclassic in a region and landscape between the interior uplands and coastal lowlands.

Finally, wetlands are of major importance for the diversity of resources they provide for human populations through their ecosystem services and functions, and their role in regulating hydrological and climatic dynamics (76). These wetlands and their agroecosystems, however, are particularly sensitive to societal and climate drivers (77). As more wetlands are lost to modern development (32), we are also losing untold examples of Indigenous knowledge and persistence. Multidisciplinary research can uncover this, as we have here, to show human ingenuity to adapt to past and future environmental changes, and we call for greater recognition and conservation of these and other cultural and natural heritage wetland sites. Indeed, this site—a rare example of ancient Maya wetland settlement to show so much preserved wood construction and evidence for human resilience through past collapse—lies amid a zone of modern intensive farming with active drainage, bulldozing, and burning.

## Materials and Methods

### Excavation.

Despite the considerable geoarchaeological, paleoecological, remote sensing, and hydrological investigations conducted over the last decade on the BOP wetland fields (13, 19–22, 25, 75), previous studies uncovered insufficient material culture to allow integrating the wetland fields within a larger framework of Maya domestic life. To understand this broader scenario, our excavations sought to establish the chronology of construction of the BOP-N settlement and identify resource acquisition strategies in situ, the chronological and spatial relationship between the settlement and the surrounding wetland field complexes, and the relationship between the settlement’s occupation to contemporaneous social, environmental, and climate changes. Thus, we devised an integrative excavation methodology that incorporated both geoarchaeological and traditional archaeological approaches. At the causeway and midden area, we prioritized exposing stratigraphy and collecting samples for characterizing and dating distinct sediment layers. At Structure 1, we maintained a higher degree of stratigraphic control by excavating at 10-cm intervals to expose anthropogenic layers, activity zones, and architectural changes, and by conducting systematic sieving of matrix material. From all units, we collected sediment samples at consistent intervals, including organic materials from well-constrained contexts for dating and shipped these to the USDA-permitted Soils and Geoarchaeology Laboratory at UT Austin for analysis. As discussed in the text, we conducted artifact analyses and counts of ceramic, lithic, and faunal assemblages.

### Radiocarbon.

We integrated the new radiocarbon determinations (*n* = 13) from the BOP-N settlement area with those from the broader BOP wetland landscape (*n* = 41) by constructing a Bayesian model (BZBOP25) in OxCal v4.4using the IntCal20 atmospheric calibration curve (78) (full details of the model are presented in *SI Appendix*). We used the Boundaries functionality in OxCal to estimate the age-range for the start and end of Maya occupation in the BOP wetlands. All radiocarbon results presented hereafter are modeled highest posterior density (HPD) 95.4% calendar age ranges (Fig. 7 and Dataset S1).

### Wood Identification.

In our 26 m^2^ surface area of excavations on Structure 1 in 2018 and 2019, we uncovered a total of nine wooden posts, two lying horizontally (PW19-F and -G) and seven vertically (PW18-1; PW19-A through - E, -H). David Lentz (University of Cincinnati) conducted Scanning Electronic Microscopy (SEM) identification of wood tissue structure from nine of these posts (Fig. 5 and *SI Appendix*, *Supplementary Note* 5). Additionally, the 2017 midden excavation uncovered one upright wooden post.

## Supplementary Material

Appendix 01 (PDF)

Dataset S01 (XLSX)

Dataset S02 (XLSX)

## Data Availability

Previously published data were used for this work (Original data. This manuscript features geochemistry, radiocarbon data, ceramic and lithics identification, and taxonomic identifications of ancient wood and faunal remains. All the data are featured in *SI Appendix* document. Geochemistry data: *SI Appendix*, Table S5. Radiocarbon data: *SI Appendix*, Tables S2 and S4. Ceramic identifications: *SI Appendix*, Figs. S1 and S2. Lithic identifications: *SI Appendix*, Fig. S3. Wood taxonomic identifications: Fig. 6 in Manuscript. Faunal taxonomic identifications: *SI Appendix*, Table S3. Previously published data. This manuscript features reanalyzed radiocarbon dates (n = 30) previously published in Beach et al. (20) (n = 26 dates) and Krause et al. (13) (n = 4 dates). These dates were reanalyzed with additional dates recently obtained (n = 14) as part of the Bayesian statistical model developed for this paper. Citations of previously published dates are included in *SI Appendix*, Table S2 under the “Reference” column, and each date in *SI Appendix*, Table S4 now has a color-coded asterisk that matches its source. Additionally, Figs. 1 and 3 derive from the LiDAR and stratigraphic data previously published in these articles. All images have been reworked to present new interpretations and have been cited inside the manuscript.). All other data are included in the manuscript and/or supporting information.
