# Supplementary material for: Maya Postclassic persistence in the Birds of Paradise Wetland Fields, Belize
Source: Proc Natl Acad Sci U S A. 2026 Mar 2;123(10):e2521892123. doi: 10.1073/pnas.2521892123 (PMC12974463; doi:10.1073/pnas.2521892123)
Supplement: Supplementary file 1 — Appendix 01 (PDF) [file pnas.2521892123.sapp.pdf]

Sánchez-Morales et al. “Maya Postclassic Persistence in the Birds of Paradise Wetland Fields, Belize.”

PNAS. Supplementary Information.

Supplementary Table 1. Cultural periods of the lowland Maya, Mesoamerica (Modified from Cook et al., 2022).

**Period name**		**Start**	**End**
Contact/Historic		After CE 1519	
Postclassic	*Late*	CE 1200	CE 1519
	Early	CE 1000	CE 1200
Classic*	*Terminal*	CE 750—850	CE 920—1000
	Late	CE 500	CE 750
	Early	CE 250	CE 500
Preclassic (Formative)	*Late*	400 BCE	CE 250
	Middle	1300 BCE	400 BCE
	Early	5500 BCE	1000 BCE
Archaic		8000 BCE	5000 BCE
Paleoindian		12,500 BCE	8000 BCE

* There is variable timing across the Maya Lowlands for the Late/Terminal Classic.

Supplementary Table 2. Recalibration of previously published radiocarbon dates on Postclassic-historical age wood artifacts from BOP region, Northern Belize, using IntCal20 (Reimer et al., 2020).

**Lab ID**	**Radiocarbon date (BP)**	**Description**	**Median calibrated age (CE)**	**Calibrated age range (CE) 95%**	**Reference**
Beta-250847	980+/-40	Charred organic matter from BOP-9	1090	990—1160	Beach et al. (2019) PNAS
Beta-207552	740+/-40	Adzed wood log	1270	1220—1385	Beach et al. (2019) PNAS
Beta-219413	170+/-40	Carved digging stick	1780	1655—Modern	Beach et al. (2019) PNAS

Supplementary Table 3. Faunal remains recovered from the 2019 excavation of the BOP-N Structure 1. The number of identified specimens (NISP) was recorded after Lyman (2008) and percent NISP (%NISP) was calculated by dividing the NISP of each taxon by the total site NISP.

Scientific Name	Common Name	NISP (%)
Dasypus novemcinctus	Nine-banded armadillo	53
Dasyprocta punctata	Central American agouti	17
Cuniculus paca	Lowland paca	24
Caviomorpha	Agoutis and pacas	40
Canis lupus familiaris	Domestic dog	6
Urocyon cinereoargenteus	Gray fox	1
Canidae	Dogs and foxes	2
Procyon lotor	Raccoon	1
Tapirella bairdii	Baird's tapir	3
Tayassuidae	Peccaries	7
Mazama sp.	Brocket deer	10
Odocoileus virginianus	White-tailed deer	16
Mammalia, Large	Mammal, deer or peccary size	18
Mammalia, Medium-Large	Mammal, deer or dog size	65
Mammalia, Medium	Mammal, dog or raccoon size	34
Mammalia, Small-Medium	Mammal, dog or armadillo size	4
Mammalia, Small	Mammal, armadillo or paca/agouti size	26
Mammalia, micro	Mammal, rat or mouse size	6
**Total Mammals**		**333**
Meleagris sp.	Turkeys	4
Galliformes	Turkeys, quails, guans	5
Crax rubra	Great curassow	6
Aves	Unidentified birds	168
Aves, medium-large	Unidentified birds (size of turkey)	47
Aves, medium-small	Unidentified birds (size of duck or hawk)	12
Aves, small	Unidentified birds (size of quail or dove)	3
**Total Birds**		**245**
Trachemys venusta	Mesoamerican slider	2
Dermatemys mawii	Central American river turtle	41
Dermatemys or Trachemys	River turtle or slider	2
Staurotypus triporcatus	Mexican musk turtle	19
Kinosternidae	Mud turtles	21
Testudines	Unidentified turtles	34
Ctenosaura (c.f.) *similis*	Black spiny-tailed iguana	1
Iguanidae	Iguanas	2
Reptilia	Unidentified reptiles	5
Total reptiles		127
Actinopterygii	Bony Fish	122
Unidentifiable Vertebrates		814
Pachychilus glaphyrus	Jute snails	234
Pachychilus (c.f.) *glaphyrus*	Jute snails	192
Pachychilus indiorum	Jute snails	110
Euglandina sp.	Wolf snail or marauder snail	4
Pomacea flagellata	Central American apple snail	8
Unidentifiable molluscs		5
**Total Invertebrates**		**553**
**Assemblage Total**		**2194**

Supplementary Figure 1. Identifiable ceramics from BOP-N Structure 1 excavations. Type: Pozo Unslipped (bowl). Identifications performed by project ceramicist Colleen Hanratty.

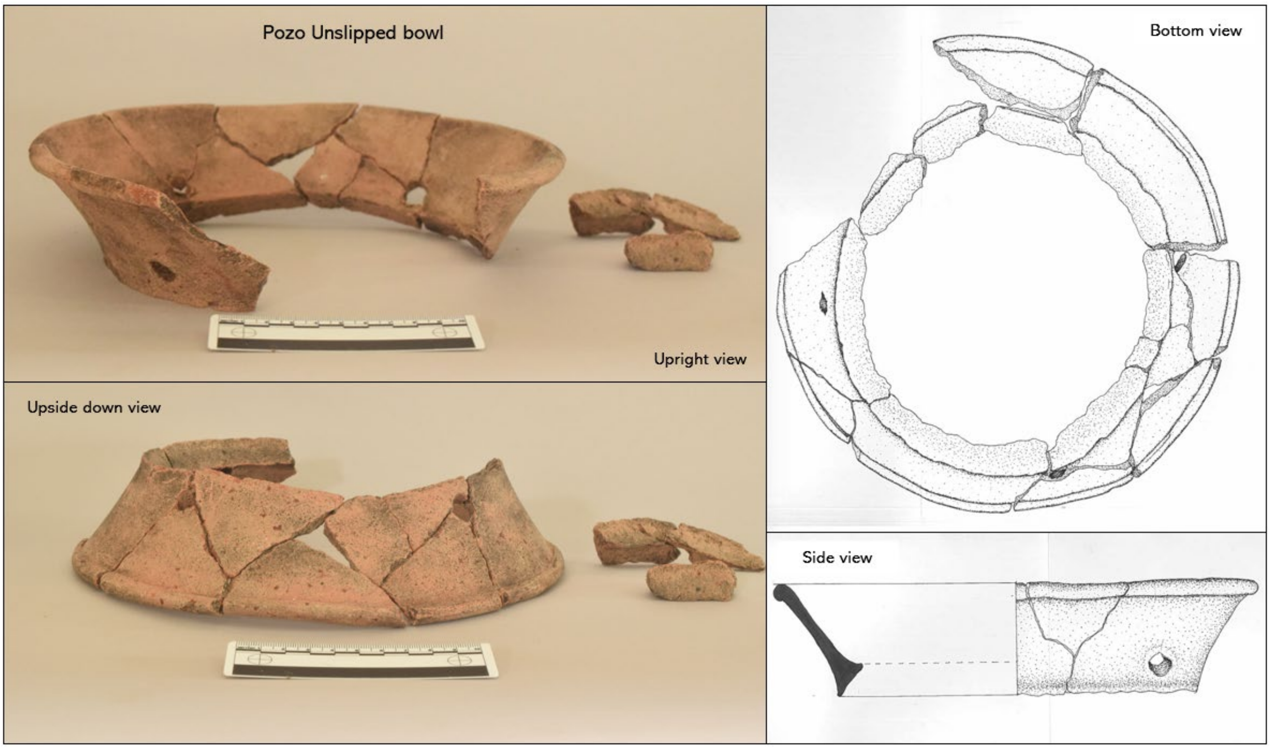

Supplementary Figure 2. Identifiable ceramics from BOP-N Structure 1 excavations. Type: Zakbeeb Incised: Variety Unspecified (bowl). Identifications performed by project ceramicist Colleen Hanratty.

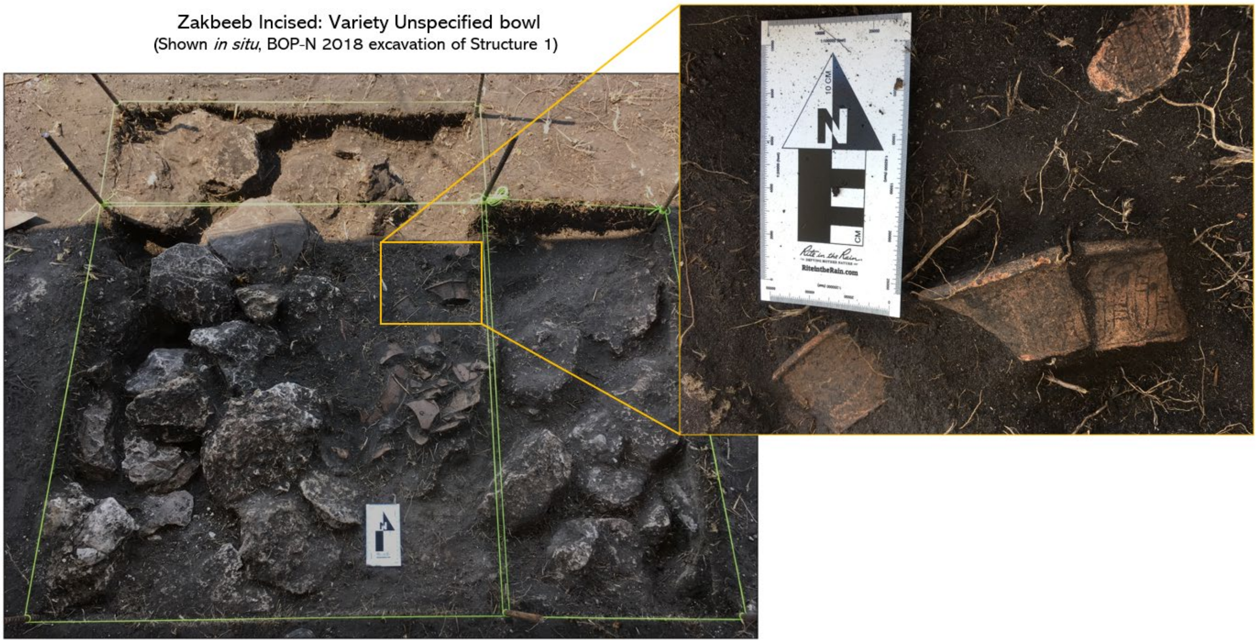

Supplementary Figure 3. Identifiable ceramics from BOP-N Structure 1 excavations. Type: Chen Mul Modeled (censer). Identifications performed by project ceramicist Colleen Hanratty.

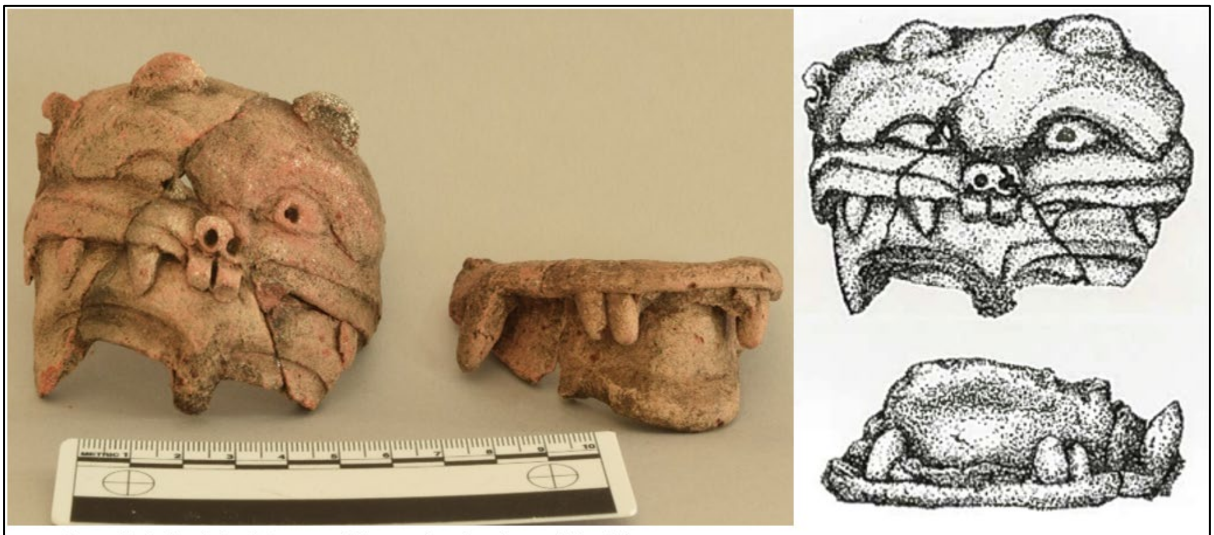

Chen Mul Modeled Censer (illustration by Jenny Martin).

Supplementary Figure 4. Selected obsidian blades from the BOP-N 2017 Mound A excavations. Identifications made by lithicist Josh Kwoka.

Obsidian blades from Mound A (BOP-N-2017).

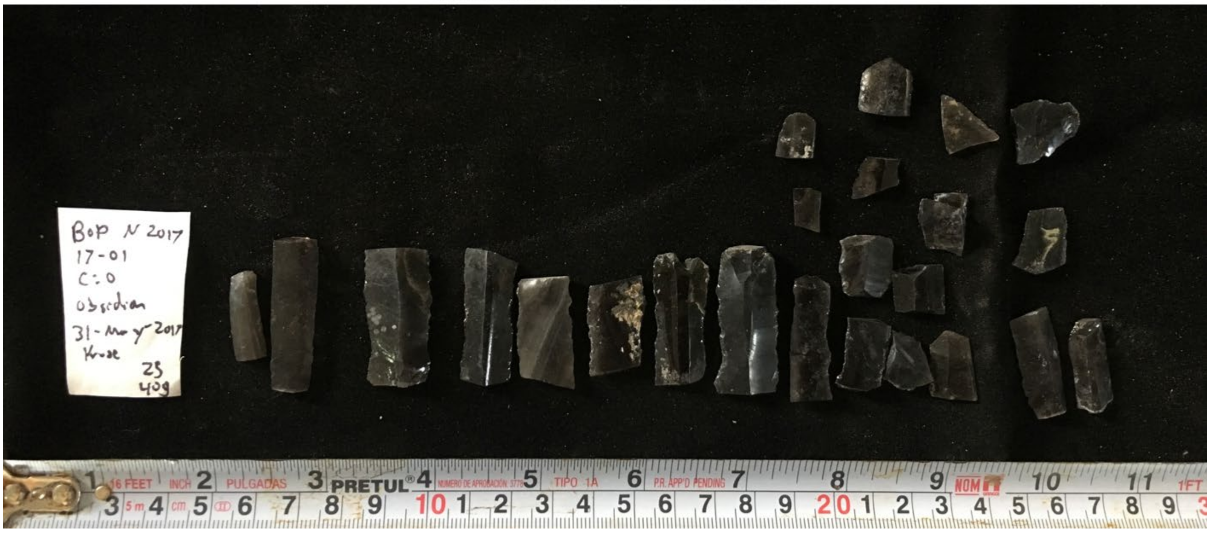

Supplementary Figure 5. Selected obsidian blades and other tools from the BOP-N 2019 excavations of Structure 1. Identifications made by lithicist Josh Kwoka.

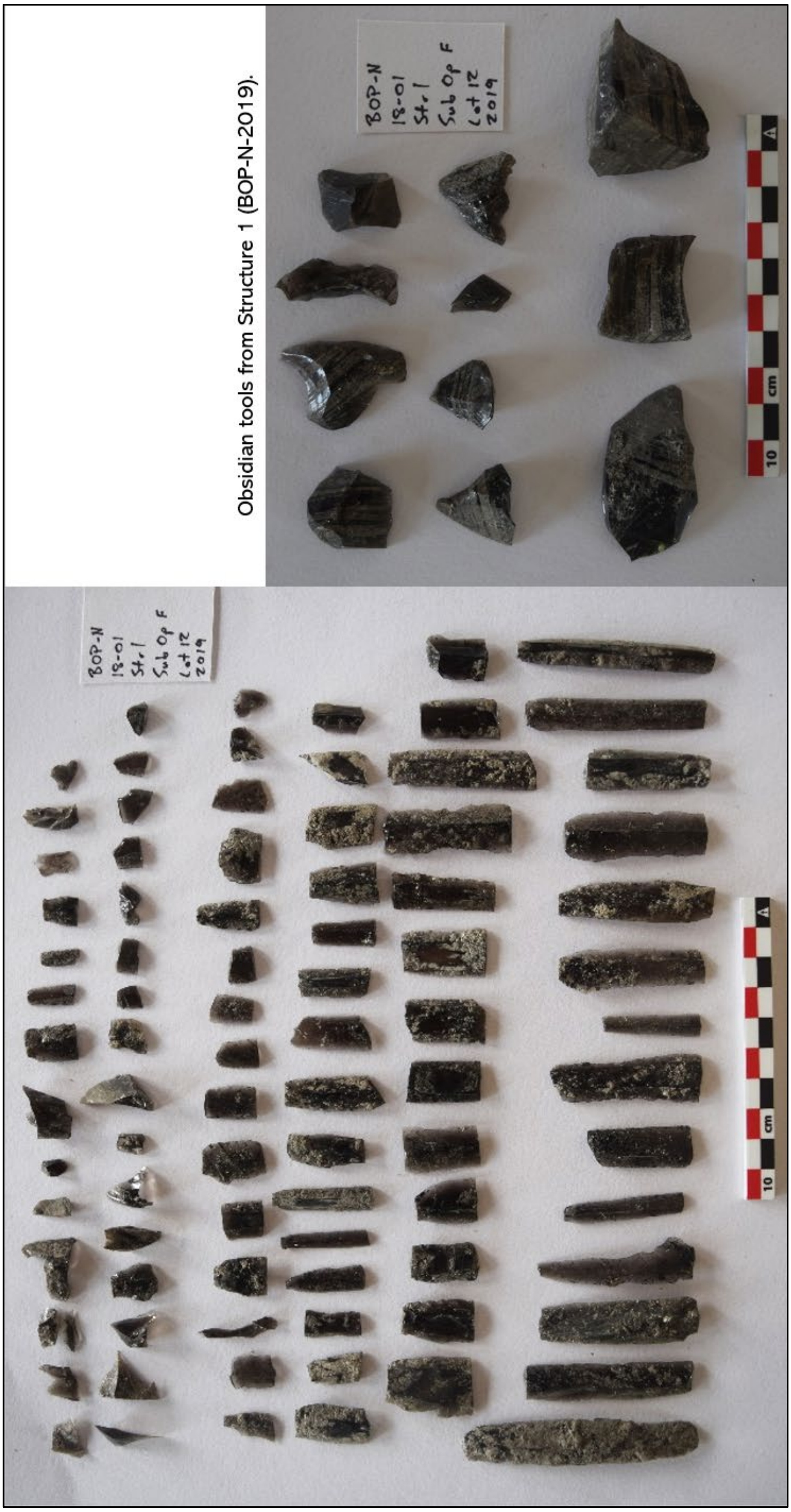

Supplementary Note 1: Details of radiocarbon dating and Bayesian modelling of samples from the BOP wetlands

1. **Treatment of previously published radiocarbon data**

All previously published radiocarbon determinations discussed in this paper have been recalibrated using OxCal version v 4.4[4] and 100% of the IntCal20 Northern Hemisphere curve[2] (Dataset S1).

2. **Radiocarbon dating the Birds of Paradise wetlands, Northern Belize**

In this study, we employed radiocarbon dating to estimate the timing of human occupation in the BOP wetlands of northern Belize. This involved collecting and dating unidentified macroscopic charcoal and short-lived plant materials (e.g. leaves) and peat from sedimentary sequences with evidence of past human activity and from archaeological strata at ancient occupation sites. We also sampled large ancient wood pieces (logs) used by the Maya in construction at the BOP-N Settlement site in the NE corner of the BOP wetlands. The dating of charcoal and plant materials aims to estimate the timing of deposition of anthropogenic sediments and stratum, while the dating of the archaeological wood is to estimate when this wood was used for construction in the settlement. All radiocarbon determinations are utilised in a single OxCal model (BZBOP25) that was designed to describe the chronology of wetland occupation by the Maya.

Samples of macropscopic charcoal and plant material were collected from archaeological strata, wrapped in aluminium foil, then bagged for storage and transportation to our laboratories. Prior to submission for dating, we examined charcoal fragments for any soil or other extraneous materials, and these were removed. Each sample was also examined for the presence of mould. Clean, mould-free samples were supplied to two facilities (Beta Analytic, Inc and International Chemical Analysis, Inc. (ICA)) for radiocarbon determinations. Sampling and laboratory procedures for radiocarbon dates used in this study, but from previous research projects, may be found in their respective publications.

We collected samples from multiple wood posts unearthed in the BOP-N-19 excavation, including Post-A (*Krugiodendron ferreum*), Post-B (*Exothea diphylla*), Post-G (*Krugiodendron ferreum*), and Post-H (*Exothea diphylla*) (Fig. 6). These tropical hardwood species are considered to be slow-growing, very dense, and often long-lived (e.g., Turner, 2001)[5] though there is scant data available on these two particular wood types. We collected small (>5 g) samples of the outer-most wood layers (sapwood, and possibly some bark) to a depth of >c. 1cm, in the field, leaving the posts *in situ*. Each wood sample was stored in labelled sample bags and transported to the Soils and Geoarchaeology laboratory at The University of Austin at Texas (USA), where they were further sub-sampled, photographed, and then submitted for radiocarbon dating.

3. Dating macroscopic charcoal, plant material, and archaeological wood specimens

The phenomena of 'old wood' effects in archaeological dating are well known. In Mesoamerican archaeology, charcoal pieces substantially older than their stratigraphic deposition dates can occur via the use of older wood fragments, burnt and unburnt, in fill materials in new constructions, or perhaps by the reuse of older wood material in later periods of construction, particular at sites with substantial, large monumental constructions that require substantial rubble and fill as a core (in the Maya world, see, for example, Arroyo and Henderson, 2020,[6] Inomata et al., 2017,[7] Inomata et al., 2022,[8] Pendergast, 2000[9]). Kennett et al. (2002)[10], working in Peru, identified multi-centennial differences in age estimates on wood charcoal versus shell from the same stratum, which they explained as the burning of wood that was already 'old' when it was incinerated. While this issue is commonplace in more arid climates, we know that ancient wood is very poorly preserved in tropical environments, and it is much less likely again that ancient wood is preserved for such long periods that it can be reused, including for burning for charcoal. At the BOP-N Settlement, some evidence that this is likely the case comes from the 14C dates on wood pieces we sampled, and that these appear in stratigraphically coherent order (that is, the underlying strata are somewhat older than the wood posts,

and that the subsequent strata that accumulated once the wood was used are younger than the wood posts) (Fig. 7). There is also the absence of any major (e.g. monumental) constructions in the BOP-N Settlement, so we find no archaeological evidence for ancient reuse or recycling of older archaeological wood.

Wood sampled from slow-growing or long-lived wood species can have an “inbuilt” age (IA) that is related to the wood itself, rather than its history of use. With slow-growing species, there is potentially up to several centuries difference in age, depending on where on the wood is sampled, due to the difference between the first years of growth (the pith, and then heartwood) and the sapwood, which, as the youngest wood, can be used to provide a terminus age estimate (that is, the radiocarbon date made on the most recent wood growth equates to when the tree stopped growing, due to being cut down). The wood in this study, *Krugiodendron ferreum* and *Exothea diphylla,* are generally considered to be slow-growing tree species (e.g. Meerow et al., 2001[11]; Colón et al., 2011[12]). However, we note that descriptions of “slow growing”, in the context of these tropical species, do not have precise definitions (e.g. cm per year) and such classifications are often relative, qualitative, and based on individual observations or experience (Brock et al. 2012[13] but also see McLaren et al. 2011[14] regarding *K. ferreum*). Regardless, given that growth rates are likely to be slow, we only sampled material from the outermost wood to a depth of c. 1 cm. In doing so, the wood submitted for dating should be very close to a terminus age for when the trunk was felled for use, limiting the amount of possible IA. Assuming ‘slow’ growth rates of 0.5 to 1.0 mm per year for tropical hardwood species used by the pre-Columbian Maya (Ralph, 1965[15]; Kennett et al., 2013[16]; Lentz and Hockaday, 2009[17]), the period of time represented by c. 1 cm of wood growth for the wood posts sampled in the BOP-N Settlement is no more than c. 10-20 calendar years. This age range might be somewhat smaller when analyzing the outermost section of a tree, where tree rings tend to have a larger width compared to heartwood bands.[15] In this study, where we have sampled the c. 1 cm of outermost material from the logs, this means that the radiocarbon accumulation of approximately up to 20 years of tree growth has probably been averaged as part of the analysis.

4. OxCal model structure and performance

We used OxCal online software 4.4 (version 173) to construct a single model for human occupation across the BOP wetlands that consists of two overlapping sequences of dates (BZBOP2025). The first sequence contains all radiocarbon dates from previous investigation of human occupation across the BOP wetlands region (“BOP Peripheral Sites”), while the second contains the 14C dates produced from the BOP-N Settlement.

Most of the radiocarbon determinations in the BZBOP2025 model were made on macroscopic charcoal, and a smaller amount made on short-lived plant material. Possible multiple sources of IA in the unidentified charcoal samples were accommodated using the Charcoal Outlier Model developed in Oxcal, implemented using the approach detailed by Bronk Ramsey (2009). We used the “general” outlier analysis (*prior probability* = 0.05) for the radiocarbon dates on the other terrestrial samples (wood) included in the model.

Initial modelling in OxCal found relatively poor model performance for the BOP Peripheral Sites sequence due to a break in chronology between dates from the Early Classic period (*n*=5) and those from the Class and Terminal Classic periods (*n*=25). Separating these dates into two separate phases resulted in a more robust model with no outlier dates detected thereafter. Initial modelling also indicated a single 14C date (ICA-19C/1269; 760 ± 30 BP) as a clear outlier, which was removed before remodelling.

The use of particular phases and sequences for the BOP-N Settlement sequence is based on stratigraphic relationships identified during fieldwork (Fig. 5), including a separate phase to describe the chronology of Maya wood use at the BOP-N Settlement site (*n*=6 dates). The final OxCal model is robust, with an A_{model} score of 94.9, an $A_{overall}$ of 93.9, and $A_{individual}$ scores typically >98 and the lowest being 71, above the minimum recommended metric of 60, though we note that OxCal A_{model} metrics are not important measures of model performance when the ‘general’ outlier model has been

employed (e.g. Weisler et al., 2024[18]). Oxcal model metrics are, therefore, no longer considered here. The unmodeled and modelled ages from the BZBOP2025 model are detailed in Dataset S1.

Supplementary Note 2.

The code for the BZBOP25 Oxcal model is reproduced below.

```
Plot()
 {
  Outlier_Model("Charcoal",Exp(1,-10,0),U(0,3),"t");
  Outlier_Model("General",T(5),U(0,4),"t");
  Curve("intcal20.14c");
  Sequence("BOP Peripheral Sites")
  {
   Boundary();
   Phase("BOP Early Classic")
   {
    R_Date("AA96037", 2066, 35);
    R_Date("ICA-19C/01102", 1830, 30)
    {
     Outlier("Charcoal", 1);
    };
    R_Date("ICA-18C/0117", 1660, 30)
    {
     Outlier("Charcoal", 1);
    };
    R_Date("ICA-18C/0116", 1770, 30)
    {
     Outlier("Charcoal", 1);
    };
    R_Date("Beta-390377", 1690, 30);
    R_Date("ICA-14C-7668", 1560, 40);
   };
   Boundary();
   Boundary();
   Phase("BOP Classic to Postclassic")
```

```
{
R_Date("ICA-19C/01101", 1410, 30)
{
 Outlier("Charcoal", 1);
};
R_Date("AA92890", 1338, 35)
{
 Outlier("Charcoal", 1);
};
R_Date("Beta-250846", 1330, 40)
{
 Outlier("Charcoal", 1);
};
R_Date("AA96036", 1283, 35);
R_Date("AA92889", 1263, 35)
{
 Outlier("Charcoal", 1);
};
R_Date("ICA-19C/01100", 1250, 30)
{
 Outlier("Charcoal", 1);
};
R_Date("Beta-250845", 1220, 40)
{
 Outlier("Charcoal", 1);
};
R_Date("ICA-18P/1225", 1210, 30);
R_Date("ICA-18P/1223", 1190, 30);
R_Date("Beta-234153 ", 1190, 40)
{
 Outlier("Charcoal", 1);
};
R_Date("ICA-17W/1205", 1170, 30)
{
```

```
 Outlier("Charcoal", 1);
};
R_Date("ICA-18P/1224", 1150, 30);
R_Date("Beta-207553", 1150, 40)
{
 Outlier("Charcoal", 1);
};
R_Date("Beta-234152", 1100, 40)
{
 Outlier("Charcoal", 1);
};
R_Date("ICA-17C/1204", 1090, 30)
{
 Outlier("Charcoal", 1);
};
R_Date("Beta-431704", 1080, 30);
R_Date("ICA-18C/0118", 1050, 30)
{
 Outlier("Charcoal", 1);
};
R_Date("Beta-250847", 980, 40)
{
 Outlier("Charcoal", 1);
};
R_Date("Beta-207551", 940, 40)
{
 Outlier("Charcoal", 1);
};
R_Date("Beta-234151", 940, 40)
{
 Outlier("Charcoal", 1);
};
R_Date("Beta-214248", 930, 40)
{
```

```
  Outlier("Charcoal", 1);
 };
 R_Date("Beta-219699", 930, 40)
 {
  Outlier("Charcoal", 1);
 };
 R_Date("Beta-240355", 830, 40);
 R_Date("Beta-431702", 790, 30);
 R_Date("ICA-18C/0120", 740, 30)
 {
  Outlier("Charcoal", 1);
 };
};
Boundary();
};
Sequence("BOP-N Settlement")
{
Boundary("Start");
Sequence("Pre-Wood Strata")
{
 R_Date("ICA-14C-7084", 1500, 30);
 R_Date("ICA-14C-7082",1250,30);
 R_Date("ICA-14C-7083",1150,30);
 R_Date("ICA-18C/0123",1040,30)
 {
  Outlier("Charcoal", 1);
 };
 First();
 Last();
 Span();
};
Phase("Wood Posts")
{
 R_Date("ICA-19W/01104",1040,30)
```

```
 {
  Outlier("General", 0.05);
 };
 R_Date("ICA-14C-6381",1030,30)
 {
  Outlier("General", 0.05);
 };
 R_Date("ICA-19W/01103",1010,30)
 {
  Outlier("General", 0.05);
 };
 R_Date("ICA-14C-6380",950,30)
 {
  Outlier("General", 0.05);
 };
 R_Date("ICA-14C-6382",910,30)
 {
  Outlier("General", 0.05);
 };
 R_Date("ICA-14C-6379",880,30)
 {
  Outlier("General", 0.05);
 };
 First();
 Last();
 Span();
 BCAD=TRUE;
};
Sequence("Postclassic Settlement I")
{
 R_Date("ICA-19C/1270",850,30)
 {
  Outlier("Charcoal", 1);
 };
```

```
  R_Date("ICA-18C/0122",820,30)
  {
   Outlier("Charcoal", 1);
  };
 };
 Sequence("Postclassic Settlement II")
 {
  R_Date("ICA-19C/1271",630,30)
  {
   Outlier("Charcoal", 1);
  };
  First();
  Last();
  Span();
 };
 Boundary("End");
 First();
 Last();
 Span();
};
};
```

Supplementary Note 3. Background details about previous investigations and context of the Birds of Paradise wetland agroecosystem.

Regional context. Wetlands are of major importance for the diversity of resources they provide for human populations through their ecosystem services and functions, and their role in regulating hydrological and climatic dynamics.[19] These wetlands and their agroecosystems, however, are particularly sensitive to societal and climate drivers.[20] As more wetlands are lost to development,[21] we are also losing untold examples of Indigenous knowledge and persistence. Multidisciplinary research can uncover this, as we have here, to show human ingenuity to adapt to past and future environmental changes, and we call for greater recognition and conservation of these and other cultural and natural heritage wetland sites. Indeed, this site—the first ancient Maya wetland village to show so much preserved wood construction and evidence for human resilience through past collapse—lies amid a zone of modern intensive farming with active drainage, bulldozing, and yearly burning.

Here we show the expanded footprint of Maya landscapes and widespread agriculture that surrounds urban centers. Most critically, this site is situated in a transition zone at the foot of the EIR, which experienced cultural florescence during the Classic but abandonment in the Postclassic, and the eastern coastal plains, where some trade and power continued and

redeveloped during the Postclassic.[22] The BOP village stands as an example of community persistence, resilience, and sustainability against the headwinds of broader societal transformation.[23] This evidence includes a new Bayesian radiocarbon chronology based on 14 previously unpublished radiocarbon dates and 30 remodeled dates from across the BOP landscape, along with evidence for site use through ceramic, lithic, and faunal assemblages that trace regional and long-range connectivity and sedimentological analyses that allow us to reconstruct the site's formation history.

Wetland field complex. BOP is the largest mapped and ground-verified contiguous system of ancient Maya wetland canals and fields, spanning 5 km^2 with another 8 km^2 nearby.[24] The complex is located in northern Belize. This perennially moist riparian system lies mostly in the Rio Bravo Conservation and Management Area, a conservation area that preserves wetlands, tropical forests, and cultural heritage in the face of rapid, regional wetland losses and deforestation.[21] Our knowledge, to date, of these ancient Maya wetland agroecosystems comes from high resolution lidar (laser imaging, detection, and ranging) mapping, more than 20 excavations of long-buried artificial canals and fields, radiocarbon dating, artifacts, elemental and isotopic geochemistry, water chemistry, pollen, macrobotanical, charcoal, and phytolith analysis.[25]

Earlier wood. Postclassic evidence for human activity in the BOP fields has been patchy in earlier excavations. Key evidence so far has come from dated sequences with micro- and macroscopic evidence of past crops, sedimentary charcoal, and carbon isotopic evidence of C_4 plant abundance in a naturally C_3 plant dominance swamp forest (i.e. isotopic C signatures of maize and other possible C4 cultivars).[25,26] We include the BOP sites 2 and 9 in our Bayesian model because they provide evidence of the region's first buried, preserved wood artifacts, located at the intersection of two wetland canals identified prior to any lidar surveys.

Supplementary Note 4. Additional details on the faunal analysis and data from Structure 1.

Faunal remains included a total of 1,641 vertebrates and 553 invertebrates. Previous work at distant parts of BOP's canals and fields reported faunal evidence from turtles, molluscs, and deer, and here we provide the first evidence of faunal materials from BOP mounds. The invertebrate assemblage (Supplementary Table 3) is dominantly Jute snails (*Pachychilus* sp.) with Wolf or Marauder snails (*Euglandina* sp.) and apple snails (*Pomacea* flagellate), and significant amounts of Jute specimens show signs of spire lopping, with the most common locations occurring at the third, fourth, or fifth whorl.

Supplementary note 5. Additional details on the hardwood species identified in Structure 1.

At the BOP-N platform, *K. ferreum* and *E. diphylla* are both present as structural building materials and possibly as tools. *E. diphylla* ("uayamcox", "culinche", or "esculinche") primarily grows in wetter areas such as floodplains and *bajos* with gleyed soils (see ref. 27, p. 89-90), whereas *K. ferreum* ("black ironwood," "chintok," or "chim tok" in Yucatec Mayan) prefers seasonally dry areas and lithosols along plains and steep slopes.[28] The species is well known for its construction and medicinal uses throughout the Circum-Caribbean and Mesoamerican regions and clearly also resists decomposition in this environment.[29] Ironwood grows to 5-10 m high (see ref. 30, p. 581) and is well known for its use in foundational beams and door posts (see ref. 31, p. 380). In addition, previous research suggests that extracts from the leaves and bark of *K. ferreum* have antimicrobial properties and medicinal uses.[32]

References cited in the SI

1. Cook, D.E., T.P. Beach, S. Luzzadder-Beach, N.P. Dunning, B.A. Smith, Long-term geomorphic change in the Maya lowlands of Central America. In: Clague J, Hardin C (eds) *Treatise on Geomorphology*, 2nd edn, vol 9. Academic Press, New York, pp 504–546 (2022).
2. Reimer, P., W. Austin, E. Bard, et al. The IntCal20 Northern Hemisphere radiocarbon age calibration curve (0–55 cal kBP). *Radiocarbon* 62(4): 725–757 (2020).
3. Lyman, R.L. *Quantitative Paleozoology.* Cambridge University Press, Cambridge (2008).
4. Bronk Ramsey, C. Dealing with outliers and offsets in radiocarbon dating. *Radiocarbon 51*(3): 1023–1045 (2009).
5. Turner, I.M. *The ecology of trees in the tropical rain forest*. Cambridge University Press (2001).
6. Arroyo, Barbara, and Lucia Henderson, 'The Monumental Aquascape of Kaminaljuyu: Water in the Archaeology of an Early Highland Site', in Brett A. Houk, Barbara Arroyo, and Terry G. Powis (eds), *Approaches to Monumental Landscapes of the Ancient Maya* (Gainesville, FL. (2020), pp. 131–151.
7. Inomata, T., Triadan, D., MacLellan, J., Burham, M., Aoyama, K., Palomo, J.M., Yonenobu, H., Pinzón, F. and Nasu, H., High-precision radiocarbon dating of political collapse and dynastic origins at the Maya site of Ceibal, Guatemala. *Proc. Natl. Acad. Sci.* 114(6): 1293–1298 (2017).
8. Inomata, T., A. Sharpe, J.M. Palomo, F. Pinzón, H. Nasu, D. Triadan, B.J. Culleton, D.J. Kennett, Radiocarbon dates of burials from Ceibal and other Pasión Maya sites, Guatemala, and the examination of freshwater reservoir effect through diet reconstruction. *Journal of Archaeological Science: Reports* 44: 103506 (2022).
9. Pendergast, D. M. "The problems raised by small charcoal samples for radiocarbon analysis." *Journal of Field Archaeology* 27(2): 237–239 (2000).
10. Kennett, D.J., B.L. Ingram, J.R. Southon, K. Wise, Differences in 14C age between stratigraphically associated charcoal and marine shell from the Archaic period site of Kilometer 4, southern Peru: old wood or old water? *Radiocarbon* 44(1): 53–58 (2002).
11. Meerow, A.W., H.M. Donselman, T.K. Broschat, *Native Trees for South Florida*. Gainesville, FL: Florida Cooperative Extension Service, Institute of Food and Agricultural Sciences, University of Florida (2001).
12. Colón, S.M., A.E. Lugo, O.M.R. González, Novel dry forests in southwestern Puerto Rico. *Forest Ecology and Management* 262(2): 170–177 (2011).
13. Brock, F., Ostapkowicz, J., Ramsey, C.B., Wiedenhoeft, A. and Cartwright, C., Paired dating of pith and outer edge (terminus) samples from pre-hispanic caribbean wooden sculptures. *Radiocarbon* 54(3-4): 677–688 (2012).
14. McLaren, K.P., Lévesque, M., Sharma, C., Wilson, B. and McDonald, M.A., From seedlings to trees: using ontogenetic models of growth and survivorship to assess long-term (> 100 years) dynamics of a neotropical dry forest. *Forest Ecology and Management* 262(6): 916–930 (2011).
15. Ralph, E.K. Review of radiocarbon dates from Tikal and the Maya calendar correlation problem. *American Antiquity*, *30*(4), 421–427 (1965).
16. Kennett, D., I. Hajdas, B. Culleton, S. Belmecheri, S. Martin, H. Neff, J. Awe, H.V. Graham, K.H. Freeman, L. Newsom, D.L. Lentz, Correlating the ancient Maya and modern European calendars with high-precision AMS 14C dating. *Scientific Reports* 3(1): 1597 (2013).
17. Lentz, D.L., B. Hockaday, Tikal timbers and temples: Ancient Maya agroforestry and the end of time. *Journal of Archaeological Sci*ence 36: 1342–1353 (2009).
18. Weisler, M.I., Q. Hua, S.L. Collins, A.J. Rogers, W.P. Mendes, Dry, leeward regions support colonization period sites: Stratigraphy, dating, and geomorphological setting of one of the

earliest habitations in the Hawaiian Islands. *The Journal of Island and Coastal Archaeology* 19(3): 610–642 (2024).

19. Parmesan, C., M. D. Morecroft, Y. Trisurat, R. Adrian, G.Z. Anshari, A. Arneth, Q. Gao, P. Gonzalez, R. Harris, J. Price, N. Stevens, and G. H. Talukdarr. *Terrestrial and Freshwater Ecosystems and Their Services.* Climate Change 2022: Impacts, Adaptation and Vulnerability: Contribution of Working Group II to the Sixth Assessment Report of the Intergovernmental Panel on Climate Change. (Cambridge University Press, 2022), pp. 197–377.
20. Caretta, M.A., A. Mukherji, M. Arfanuzzaman, R.A. Betts, A. Gelfan, Y. Hirabayashi, T.K. Lissner, … S. Supratid. *Water.* Climate Change 2022: Impacts, Adaptation and Vulnerability: Contribution of Working Group II to the Sixth Assessment Report of the Intergovernmental Panel on Climate Change. (Cambridge University Press, 2022), pp. 551–712.
21. Doyle, C., T. Beach, and S. Luzzadder-Beach. Tropical Forest and Wetland Losses and the Role of Protected Areas in Northwestern Belize, Revealed from Landsat and Machine Learning. *Remote Sensing* 13(3): 379 (2021).
22. Turner, II, B.L. and J.A. Sabloff. Classic period collapse of the central Maya lowlands: Insights about human-environment relationships for sustainability. *Proc. Natl. Acad. Sci.* 109(35): 13908–13914 (2012).
23. Guderjan, T.H., S. Krause, S. Luzzadder-Beach, T. Beach, and C. Brown. *Perspectives on the Ancient Maya of Chetumal Bay* Ch. 5 (Univ. Press of Florida, 2016).
24. Beach, T., S. Luzzadder-Beach, S. Krause, T. Guderjan, F. Valdez, Jr., J.C. Fernandez-Diaz, S. Eshleman, and C. Doyle. Ancient Maya wetland fields revealed under tropical forest canopy from laser scanning and multiproxy evidence. *Proc. Natl Acad. Sci.* 116(43): 21469–21477 (2019).
25. Beach, T., S. Luzzadder-Beach, N. Dunning, J. Jones, J. Lohse, T. Guderjan, S. Bozarth, S. Millspaugh, and T. Bhattacharya. A review of human and natural changes in Maya Lowland wetlands over the Holocene. *Quat. Sci. Rev*. 28: 1710–1724 (2009).
26. Krause, S., T.P. Beach, S. Luzzadder-Beach, D. Cook, S.R. Bozarth, F. Valdez, Jr., and T.H. Guderjan, Tropical wetland persistence through the Anthropocene: Multiproxy reconstruction of environmental change in a Maya agroecosystem. *Anthropocene* 34: 100284 (2021).
27. Standley, P. and Y. J. Steyermark. Flora of Guatemala (Chicago Natural History Museum, 1949).
28. Martínez, E. and C. Galindo-Leal. La vegetación de Calakmul, Campeche, México: Clasificación, descripción y distribución. *Boletín de la Sociedad Botánica de México* 71: 7–32 (2002).
29. Balick, M. J., M. Nee, and D. E. Atha. *Checklist of the vascular plants of Belize, with common names and uses*. (The New York Botanical Garden Press, 2000).
30. Acevedo-Rodríguez, P. *Flora of St. John, U.S. Virgin Islands*. (The New York Botanical Garden Press, 1996).
31. Roys, R. L. *The Ethno-Botany of the Maya*. (Institute for the Study of Human Issues, 1976).
32. Uc-Cachón, A. H., A. de J. Dzul-Beh, G. A. Palma-Pech, B. Jiménez-Delgadillo, J. S. Flores-Guido, C. Gracida-Osorno, G. M. Molina-Salinas. Antibacterial and antibiofilm activities of Mayan medicinal plants against Methicillin-susceptible and-resistant strains of Staphylococcus aureus. *Jour. of Ethnopharma*. 279: 114369 (2021).
